# An Innovative Oral Ex Vivo Biofilm Model for Antimicrobial Investigations

**DOI:** 10.3390/pathogens15040375

**Published:** 2026-04-01

**Authors:** Stefan Kranz, Markus Heyder, André Guellmar, Michael Gottschaldt, Ulrich S. Schubert, Bettina Loeffler, Bernd Sigusch, Markus Reise

**Affiliations:** 1 Department of Conservative Dentistry and Periodontology, University Hospitals Jena, An der Alten Post 4, 07743 Jena, Germanybend.w.sigusch@med.uni-jena.de (B.S.);; 2 Laboratory of Organic and Macromolecular Chemistry (IOMC), Friedrich Schiller University Jena, Humboldtstraße 10, 07743 Jena, Germanyulrich.schubert@uni-jena.de (U.S.S.); 3 Jena Center for Soft Matter (JCSM), Friedrich Schiller University Jena, Philosophenweg 7, 07743 Jena, Germany; 4 Institute of Medical Microbiology, Jena University Hospital, Friedrich Schiller University of Jena, 07743 Jena, Germany; bettina.loeffler@med.uni-jena.de

**Keywords:** antimicrobials, chlorhexidine, disinfectant, oral biofilm, periodontitis, subgingival biofilm

## Abstract

The methodical work describes all the necessary steps for establishing a stable oral ex vivo biofilm using saliva and crevicular plaque samples from periodontal healthy donors. First, cover slips were preconditioned with saliva supernatants and subsequently inoculated with crevicular plaque suspensions. Ex vivo biofilm formation was characterized by confocal laser scanning microscopy (cLSM) after 1, 4, 24, 48 and 72 h of anaerobic cultivation. Exemplarily, the inhibitory characteristics of blackcurrant fruit extracts [all-fruit juice (AFJ); alcoholic fraction from berry skins (AFBS)] were observed on 1, 4 and 24 h-aged ex vivo biofilms. Chlorhexidine (CHX, 0.2%) served as positive control. After direct contact (3 min), biofilms were dispersed, plated onto agar and anaerobically cultivated for 24 h. Early ex vivo biofilms (1 h-biofilm) showed scattered microbial colonies. After 4 h of cultivation, a multilayered biofilm was formed. Biofilm mass gradually increased, displaying a complex polymicrobial structure after 24 h. At 72 h, the biofilms had a dense three-dimensional appearance. Treatment with AFJ and CHX was more efficient in inhibiting biofilm growth compared to AFBS. Early biofilms (1 h, 4 h) were more susceptible to AFJ and CHX compared to 24 h-biofilms. The introduced model can be recommended for testing the efficiency of plaque-controlling agents.

## 1. Introduction

Oral biofilms, also referred to as dental plaque, inhabit a large variety of different microbes. Besides fungi, protozoa, archaea and viruses, bacteria are the most dominant species [1]. With >700 different strains, bacteria are considered the main oral colonizers [2]. Within the biofilm structure, microbes are embedded in an extracellular polymeric matrix, which provides protection, nutrition and homeostasis [3,4].

Biofilm formation follows a distinct growth pattern, starting with the attachment of bacterial pioneer species, such as *Streptococcus gordonii*, *Streptococcus sanguinis*, *Streptococcus mitis* and *Streptococcus oralis*. This step is also mediated by components of the so called acquired pellicle, which forms on the surface of the teeth immediately after cleaning and is composed of salivary proteins, carbohydrates and lipids. Recently, we were able to demonstrate that protein adsorption is influenced by the type of underlaying substrate, which might significantly impact initial bacterial colonization, too [1,5]. Once the pioneer strains have attached, co-aggregation of other microbes leads to biofilm growth, forming a unique three-dimensional structure over time [1,6,7,8,9].

In order to control oral biofilm formation, various anti-plaque products are available on the market. These include mouthwashes, gels, foams and varnishes, containing synthetic and natural antimicrobial compounds. Prior to clinical approval, however, the suppressive activity of these products is usually tested in planktonic bacterial cell cultures and artificially grown biofilms, established from single or multiple species. However, most of these approaches, in particular those based on only mono-species cultures, do not reflect the real clinical situation where there are complex ecological interactions.

Therefore, efforts have led to the introduction of more realistic models that mimic oral biofilm formation to a higher degree [10,11]. Besides the establishment of multi-species consortia grown from unique physiological and ecological bacterial groups, such as the Marsh consortium or Zurich model, microcosms were established as well. Such microcosms are laboratory-based biofilms grown from collected clinical samples (saliva, plaque) and are characterized by a more complex ecology [12,13].

Considering these aspects, the present manuscript describes steps that are necessary for establishing an ex vivo microcosm-based oral biofilm from clinical crevicular and saliva samples. Thus, the protocol follows principal mechanisms that are fundamental for sufficient in vitro biofilm formation. The described model can be applied for studying the efficiency of biofilm controlling agents. Exemplarily, the present manuscript investigated the inhibitory effects of extracts derived from blackcurrant fruits (*Ribes nigrum*) on ex vivo biofilm growth. Additionally, the biofilm inhibitory characteristics of chlorhexidine (CHX), a common oral disinfectant was also analyzed [14,15].

As recently reported by our group, blackcurrant all-fruit juices are sufficient to suppress various oral pathogenic bacteria in the planktonic phase [16]. This behavior is due to the high content in polyphenols, which belong to the class of secondary plant metabolites and are considered strong antimicrobial active substances [17]. The efficiency of blackcurrant fruit extracts in inhibiting oral biofilm formation has not yet been observed in detail. Therefore, the introduced model was exemplarily applied for investigating the inhibitory characteristics of different blackcurrant fruits extracts as well as from CHX.

## 2. Materials and Methods

### 2.1. Ex Vivo Biofilm Model

Ex vivo biofilms were established from subgingival plaque and unstimulated whole saliva samples that were collected from periodontal healthy volunteers between 23 and 26 years of age (n = 10; 5 males, 5 females). All donors were recruited from the patient pool of the Department of Conservative Dentistry and Periodontology, University Hospital, Jena, Germany. The experimental protocol was reviewed and approved by the local Ethics Committee (Medical Faculty, University Hospital, Jena, Germany; ID: 5355-11/17). Participants showed overall good health without a history of systematic diseases, no present medication or antibiotics treatment during the past three months. Only non-smokers were included. Prior to sample collection, participants had to refrain from oral hygiene for 24 h. The applied biofilm model is based on former results published by Müller and Walker et al. [18,19] and modified as follows:

Biofilms were collected from the approximal molar or premolar region of each quadrant using sterile endodontic paper points (ISO 30). The paper points were inserted in the gingival sulcus for 10 s and immediately afterwards transferred to an Eppendorf tube containing 10 mL of Schaedler fluid media and stored at 4 °C until further processing. Additionally, 5 mL whole unstimulated salivary was collected from each participant and also stored at 4 °C until use.

All collected samples were transferred to the laboratory and immediately processed as follows. Patient specific subgingival biofilm samples were cultivated in 10 mL Schaedler fluid media under anaerobic conditions for 24 h. Afterwards the samples were centrifuged (3500 rpm, 5 min) and the obtained bacterial pellets resuspended in fresh Schaedler fluid media. The microbial inoculum was then adjusted to an optical density of OD546 nm = 0.5 (10^7^ CFU/mL).

Meanwhile, each collected saliva sample was centrifuged twice (13,000 rpm, 5 min) and the resulting supernatants (100 µL) pipetted onto coverslips placed in 24-well polystyrene cell culture plates. Subsequently, all plates were incubated at 37 °C for 2 h for pre-conditioning (pellicle-coating) the glass surfaces.

Afterwards, all coverslips were gently rinsed with PBS three times and covered with 600 µL of the respective plaque inoculum of the same donor who also provided the saliva sample. The prepared plates followed cultivation under anaerobic standard conditions. After 1, 4, 24, 48 and 72 h bacteria/biofilms were fixed in 4% paraformaldehyde solution (4% PFA/PBS) and stained with propidium iodide (10 µg/mL in PBS). Ex vivo biofilm formation was imaged using a confocal laser-scanning microscope LSM510 (Carl Zeiss, Jena, Germany) equipped with a 20× planapochromat objective. Propidium iodide labeled bacteria were excited using a helium-neon laser (543 nm). (For high-power photomicrographs, optical sections were collected using a 40× C-Apochromat water immersion objective.)

### 2.2. Inhibition of Ex Vivo Biofilm Growth by Blackcurrant Fruit Extracts

For experimental purpose, freshly harvested blackcurrant fruits (*Ribes nigrum*) grown in Thuringia, Germany were used. Deep frozen fruits (−20 °C) were transferred to the laboratory for further processing. Test solutions were prepared from the fruits by two different protocols.

### 2.3. Blackcurrant All-Fruit Juice (AFJ)

Blackcurrant fruits were processed using a conventional blender. The resulting mixture was centrifuged at 4800 rpm for 10 min, supernatant collected and centrifuged another time at 13,000 rpm for 5 min in order to remove all solid pulp and seed components. The resulting juice was then sterilized at 70 °C for 30 min and stored in 1.5 mL Eppendorf tubes at −20 °C.

### 2.4. Alcoholic Fraction from Blackcurrant Beery Skins (AFBS)

Skins from blackcurrant fruits were mixed with ethanol in a ratio of 1:3 and placed on a tumbling table at room temperature for 30 min. The aliquots were then centrifuged at 4 °C, 1500 rpm for 5 min. The resulting supernatants were collected and transferred to an open, sterile reaction vessel, placed alternately in an incubator at 37 °C following evaporation at room temperature. The cycle was terminated when all ethanol was completely evaporated from the solution. The resulting solutions were then dissolved in DMSO and aliquots deep frozen at −20 °C. For experimental purposes, the DMSO samples were dissolved in PBS up to the initial volume of the applied berry skins.

### 2.5. Inhibitory Effects of AFJ and AFBS on Ex Vivo Biofilm Growth

In order to evaluate the inhibitory characteristics of AFJ and AFBS, ex vivo biofilms were grown for 1, 4 and 24 h. Prior to direct incubation with the respective test solution, no-adherent microbial cells were removed by gently washing the biofilms once with 500 µL of PBS. Subsequently, biofilms were covered with 500 µL of the respective blackcurrant fruit extract (AFJ, AFBS). As a positive control, 0.2% chlorhexidine (CHX) was applied and treatment with PBS served as a negative control. After exposure for either 1 or 3 min, all test and control solutions were removed and the ex vivo biofilms gently rinsed twice with PBS. To verify the suppressive effects, biofilms were dispersed by rigorously pipetting for several times. Afterwards, the biofilm suspensions were serial diluted up to a factor of 10^−6^, aliquots (100 µL) plated onto Schaedler agar and incubated under anaerobic standard conditions. After 48 h of incubation colony forming units (CFU/mL) were determined.

### 2.6. Statistical Analysis

For analyzing the influence of the respective blackcurrant fruit extracts on microbial growth, a multi-factorial ANOVA was performed. This included a selection of three factors with different levels: test solution (4 levels: AFJ, AFBS, CHX, PBS); biofilm age (3 levels: 1 h vs. 4 h vs. 24 h) and exposure time (2 levels: 1 min vs. 3 min). Main effects and interactions were investigated, and corresponding post hoc *t*-tests (corrected for multiple testing using Holm correction) were applied. Intragroup comparison was additionally carried out using non-parametric Mann–Whitney-U test. The level of significance was set to α = 0.05. For statistical analysis SPSS version 30.0 was used.

## 3. Results

### 3.1. Characterization of Oral Ex Vivo Biofilms

**Figure 1:**
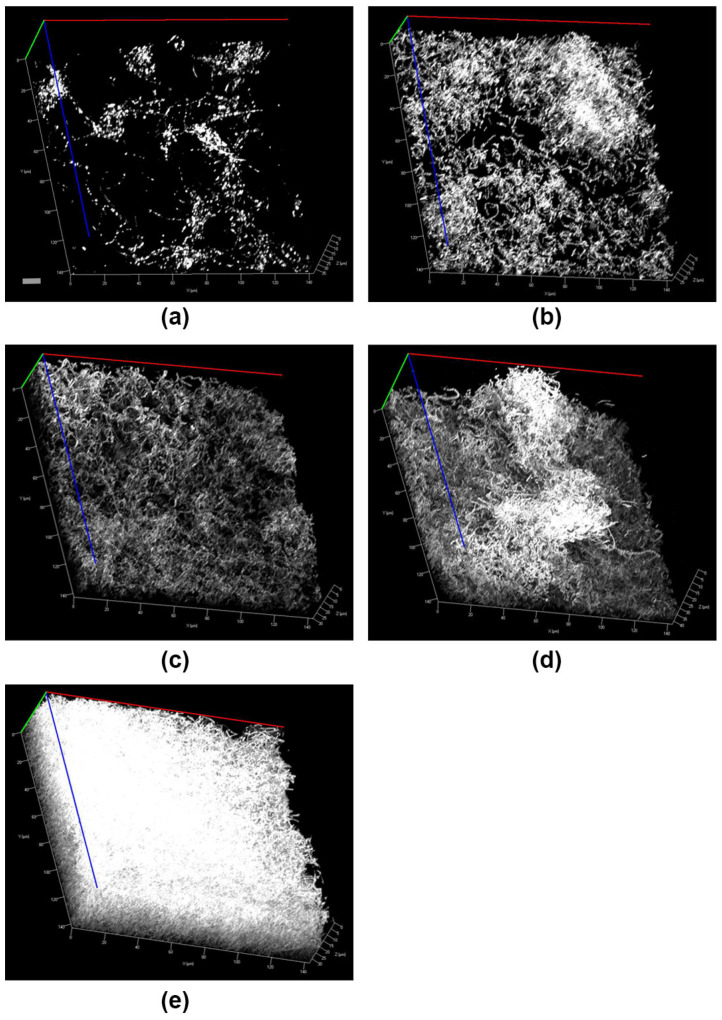
Oral ex vivo biofilm formation at different cultivation times observed by cLSM. (**a**) 1 h-biofilm, (**b**) 4 h-biofilm, (**c**) 24 h-biofilm, (**d**) 48 h-biofilm and (**e**) 72 h-biofilm. Scale bar represents 3 µm.

The different stages of ex vivo biofilm formation are summarized in Figure 1. Ex vivo biofilm growth was documented by cLSM after 1, 4, 24, 48 and 72 h. As shown, biofilm volume increases by time. After just one hour of cultivation, local microbial conglomerates are formed. These colonies appear island-like on the glass surface. Different adhering cocci- and rod-like microbes can clearly be distinguished. These initial microbial colonies start to proliferate forming continuous polymicrobial layers. After 24 h of cultivation a multilayer biofilm was established. In that time, ex vivo biofilms increased gradually in volume, forming a dense polymicrobial multilayered structure. After 72 h of cultivation, a mature ex vivo biofilm was formed.

### 3.2. Efficiency of Blackcurrant Fruit Extracts in Inhibiting Ex Vivo Biofilm Growth

As shown by the results, blackcurrant fruit extracts were able to significantly inhibit biofilm growth. It was verified that suppressive activity was stronger on initial biofilms (1 and 4 h). Significance was verified by two-way ANOVA (F (3.06, 9.0) = 7.37, *p* < 0.001).

Biofilms grown for 1 or 4 h were significantly inhibited by all tested solutions. From all blackcurrant fruit extracts, AFJ revealed the strongest inhibitory effects. In detail, direct exposure for 3 min caused suppression of the 1 h-biofilm by 1.93 log and of the 4 h-biofilm by 2.21 log, when compared to the negative control (Table 1). However, inhibitory activity was not significantly different to treatment with 0.2% CHX (positive control, *p* = 0.075).

AFBS showed only minor suppressive activity. On the 1 h biofilm, incubation of AFBS for 3 min caused suppression by only 1.0 log in CFU/mL, when compared to the negative control (PBS). In comparison, exposure of the 1 h-biofilm to 0.2% CHX (3 min) resulted in growth inhibition by 3.23 log in CFU/mL, which was also significantly different to the action of AFJ (*p* < 0.001). In the 4 h biofilm, AFBS showed only very low inhibitory activity.

On the 24 h-biofilms, only AFJ caused any significant growth inhibition. Neither incubation with AFBS nor CHX caused any significant inhibitory effects, when compared to the negative control (Table 1).

**Figure 2:**
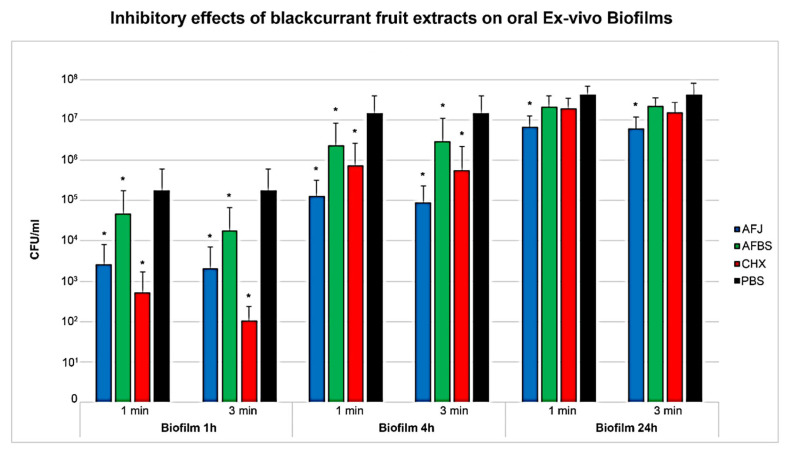
Inhibition of ex vivo biofilm growth by blackcurrant extracts. Exposure of all-fruit juice (AFJ) and alcoholic fraction from berry skins (AFBS) for 1 and 3 min caused significant inhibition of biofilm growth. Significance to the negative control (PBS, black column) is marked by star (*p* < 0.05). CHX 0.2% was used as positive control (red columns). Ex vivo biofilms grown for 1, 4 and 24 h were used for examination.

In regard to the applied exposure times significance was also reported by two-way ANOVA (F (3.0, 27.0) = 3.75, *p* = 0.023). In general, stronger inhibitory effects were observed when exposure times of 3 min were applied. However, two-way ANOVA reported no significant interaction between exposure time and biofilm age (F (1.01, 9.31) = 0.95, *p* = 0.358). All results are also summarized in Figure 2.

## 4. Discussion

The presented methodical work describes all steps that are necessary for establishing a stable ex vivo oral biofilm from crevicular plaque and saliva samples. Furthermore, the described model was exemplarily used for analyzing the antimicrobial efficiency of blackcurrant fruit extracts in inhibiting ex vivo biofilm growth.

### 4.1. Characteristics of the Ex Vivo Biofilm Model

In the present study, clinical samples from 10 periodontal healthy donors were used for growing ex vivo biofilms. The number of donors was calculated in accordance with Walker et al. who included eight individuals [19]. Using 10 donors can cause some variability, as the microbiome differs between individuals. The microbial composition of each ex vivo biofilm must be considered unique, since no pooled samples were used for inoculation. In the inhibitory assays, all results are shown as median values, calculated from the total number of ex vivo biofilms that were tested (n = 10).

Compared to already existing oral biofilm models, the described protocol has some advantages. Because samples from multiple sources (crevicular plaque, whole unstimulated saliva) were used for inoculation, high microbial diversity is likely. Many existing models only use single sources (saliva vs. plaque) which limits the established microcosm biofilms to some local representatives. In addition, the developed ex vivo biofilms include native microbes, presenting greater resistance and higher pathogenicity. This is a further advantage over biofilms established from only laboratory strains, ensuring more realistic results. Conveniently, ex vivo biofilms can also be developed from clinical samples of only one single donor without the use of extensive equipment, making the proposed model cost-effective too. The described protocol is also highly adaptive, since ex vivo biofilms from diseased patients (dysbiotic biofilms) can be developed as well (gingivitis vs. periodontitis vs. periimplantitis). Whether these biofilms react differently, still needs to be investigated in detail. Furthermore, stable ex vivo biofilms form within 24 h of cultivation, as saliva and plaque are used from the same donor. This is a further advantage over microcosm biofilms grown from pooled or single clinical sources, since these biofilms are often characterized by extended maturation times [19,20].

In the oral cavity, biofilm formation starts with the adsorption of salivary proteins, carbohydrates and lipids to the cleaned tooth surface. This layer, also known as acquired pellicle, is crucial for initiating bacterial attachment. As recently shown by our group and also confirmed by other authors, pellicle composition, especially of the protein fraction, is significantly influenced by the type of underlying material and, thus, also seems to impact initial bacterial colonization in a distinct way [1,5,19,21].

### 4.2. Surface Preconditioning Using Unstimulated Whole Cell-Free Saliva

The present protocol favors pellicle-coating with unstimulated whole cell-free saliva, instead of human serum as proposed by other authors [22,23]. Salivary proteins, such as acidic proline-rich proteins, cystatin, statherin, and S100-A9 proteins adsorb fast to the surface, while serum proteins attach more slowly [24].

Once an initial pellicle is established, single bacterial cells as well as cell aggregates adhere to the surface. This step is mediated by specific surface adhesins that bind to saccharide receptors of the pellicle layer [25,26]. However, initial microbial colonies are formed by pioneer species such as *Actinomyces* spp., *Streptococcus* spp., *Lactobacillus* spp. and *Candida* spp. [27]. This is also in line with observations of our own group [5].

The establishment of a salivary pellicle is seen as fundamental step in the described ex vivo model. Without pellicle pre-conditioning, microbes are rather unable to adhere to the applied glass surfaces, resulting in the formation of only loose microbial conglomerates. Therefore, cell-free supernatants from unstimulated whole saliva samples were used for pre-conditioning the cover slips. Interestingly, best culture results were obtained when saliva and plaque samples from the same donor were applied. Similar results have also been observed by other authors. Applying pooled saliva from multiple donors resulted in prolonged maturation times or in an early detachment of initial colonies [19,20]. However, combining saliva and plaque from the same donor is of advantage, since ex vivo biofilms form more sufficiently [19,20].

Key reasons for this specific behavior might be based on interactions between bacteria and salivary proteins. Bacteria from the same donor have already developed the ability to recognize these donor-specific proteins and bind to them efficiently [28]. Furthermore, saliva also contains agglutinins that can promote bacterial aggregation. Using plaque and saliva from the same donor facilitates bacterial aggregation [29,30]. Also, the plaque community is already specialized to break down glycoproteins that originate from the same donor. This ensures a more sufficient nutrient match compared to “unfamiliar proteins” of pooled sources (heterogeneous saliva) [31]. Another reason might also be the occurrence of competing microbes which leads to unstable community structures and antagonistic interactions, preventing coaggregation and cell attachment [32]. Also, in pooled samples, conflicting environmental signals might disrupt cell signaling (quorum sensing), resulting in insufficient bacterial communication [1,33]. Components of the adaptive and innate host defense system are also present within the saliva. These often function synergistically, and at sublethal concentrations, causing the development of complex relationships between the host and resident microbiota. Dysbiosis might then occur rapidly when salivary immune components from different individuals are combined [34].

Coating artificial surfaces with cell free human saliva for enhancing microbial attachment and biofilm formation is also proposed by other authors [18,19,35,36,37,38].

By applying the described methodology, surface-attached colonies have been observed in the present work already after 1 h of ex vivo biofilm cultivation. This confirms the efficiency of the applied technique (Figure 1a).

### 4.3. The Use of Clinical Plaque Samples

For establishing an ex vivo biofilm, dental plaque from the crevicular region was used. Artificial biofilms originating from subgingival sources are characterized by a microbial diversity that includes both Gram-positive and Gram-negative species.

In this context, the presence of *Fusobacterium* spp., in particular *Fusobacterium nucleatum* appears to play an important role in the process of biofilm formation. As known, this species possesses multivalent adhesins that creates strong connections between early and later colonizers, thus enabling the formation of a complex and durable polymicrobial biofilm structure [9,27,39,40,41]. In our opinion, the use of subgingival or sulcus plaque increases the probability of *Fusobacterium* spp. being enclosed, contributing sufficient biofilm formation under laboratory conditions [42]. This is supported by observations of our own that detected *F. nucleatum* in subgingival plaque samples collected from periodontitis diseased patients by a proportion of 77%. Using plaque from a subgingival source was efficient in growing initial biofilms [43].

By applying the described method, stable ex vivo biofilms can be established without the use of expensive equipment in a rather short time. Ex vivo biofilms can be used for investigations already after 24 to 72 h of cultivation. This is advantageous, since many other protocols need extensive equipment or biofilm formation occurs rather slowly.

### 4.4. Anti-Biofilm Characteristics of Blackcurrant Fruit Extracts

In the present work, the established ex vivo biofilms were used for examining the suppressive characteristics of blackcurrant fruit extracts on biofilm growth. As recently published by our group, blackcurrant all-fruit juice reveals significant suppressive behavior on different Gram-positive and Gram-negative oral bacteria in planktonic culture [16].

In the present investigation AFJ was more efficient in inhibiting biofilm growth compared to AFBS. The inhibitory effect of AFJ is likely more pronounced due to its higher content in polyphenols. AFBS was produced exclusively from the fruit skins, while in AFJ the seeds and pulps were also included. Quantitative analyses might have verified differences in the total polyphenolic content and should be included in future studies.

As already known, blackcurrant fruits and leaves are rich in polyphenols, especially flavonols (kaempferol, quercetin, and myricetin), their glycosides (kaempferol-3-*O*-glycoside, quercetin-3-*O*-glycoside, and quercetin-3-*O*-rutinoside), anthocyanins, and tannins. Blackcurrant anthocyanins (delphinidin-3-*O*-rutinoside and cyanidin-3-*O*-rutinoside) are considered significant antimicrobial metabolites, constituting between 60% and 85% of the total phenolic berries content [44,45,46,47]. Detailed information about the antimicrobial activity of blackcurrant components can also be reviewed at [48].

In the present study, two different extracts were prepared from blackcurrant fruits, with all-fruit-juice (AFJ) being most effective. Overall, the inhibitory activity on the ex vivo biofilm was low, since suppression did not exceed 2.21 log CFU/mL in maximum. For agents to be termed: “antimicrobial active”, a reduction of at least 3 log in CFU/mL must be given [49]. This was only the case with CHX, where a maximum inhibitory effect of 3.23 log CFU/mL was observed, but only in the initial phase of biofilm formation (1 h-biofilm). Older ex vivo biofilms were significantly less susceptible to CHX. In ex vivo biofilms grown for 4 and 24 h, treatment with AFJ was comparable to the action of CHX.

But, the inhibitory activity of AFJ and AFBS is currently of minor clinical relevance. Future investigations should focus on identifying the involved mechanisms more precisely. This might help to increase the anti-biofilm activity of extracts derived from berry fruits in general. Besides medical applications, the current results might also be interesting for anti-food-spoiling strategies.

### 4.5. Effects of CHX on Ex Vivo Biofilm Formation

While some studies confirm the efficiency of CHX in inhibiting oral biofilm formation, there are also studies reporting insufficient action, especially on mature biofilms. This is in line with observations of the present investigation. In this regard, a recent study reported from 0.1% CHX that inhibited a 7-day old microcosm biofilm by only 7.7% [50]. In another study a 24 h-biofilm composed of 14 different species was treated with 0.12% CHX for 5 min on three consecutive days. The treatment caused an initial drop in bacterial viability followed by an instant recovery to sharply 10^7^ cells/cm^2^ (similar to the concentration at 0 h) [51]. Also, the effect of various CHX-containing mouthwashes was investigated, revealing a wide variety of inhibitory effects. After single application, five of the tested mouthrinses failed to significantly inhibit biofilm growth [52].

While there is excellent antibacterial activity of CHX on free-floating bacteria, eradication of species within mature biofilms is often insufficient. The low anti-biofilm activity of CHX on mature biofilms might be caused by the extracellular matrix, acting as a physical barrier. Also, CHX might be neutralized by negatively charged molecules that are present within the biofilm (e.g., extracellular DNA). Unlike NaOCl, CHX also only has limited ability to dissolve organic material, resulting in an inefficient breakdown of matrix components to access bacteria that are located deeper within the biofilm structure. Furthermore, bacteria are also able to adapt to sublethal CHX doses by changing their cell wall structure, permeability, and efflux pump efficiency [53].

### 4.6. Inhibition of Biofilm Growth by Fruit Extracts

While there is sufficient information about the activity of CHX, results about the inhibitory activity of berry phenols and especially of blackcurrants fruit extracts are still lacking. However, most investigations focus on the anti-biofilm activity of extracts derived from grape seeds, cranberries or sour cherries [54,55].

In the case of sour cherries, efficient inhibition of salivary alpha amylase bound to biofilm bacteria was reported, thus reducing the capability to break down starch into glucose [56]. Those interactions often cause a limitation of available nutrients [57]. Furthermore, studies on cranberry extracts revealed inhibitory effects on the glucan synthesis and F-ATPase activity, influencing bacterial cell attachment and energy homeostasis [54,58,59]. Probably, the berries sugar matrix also plays a distinct role in inhibiting microbial viability. In summary, anti-biofilm mechanisms of berry polyphenols are diverse, targeting quorum sensing, enzyme activity, cell membrane functions and also bacterial attachment mechanisms [54,55].

However, for complex extracts, such as AFJ and AFBS, the mode of action involves more than immediate contact killing (e.g., interference with quorum sensing, metabolic disruption, or gradual polyphenol–membrane interactions). A 3 min incubation window may severely underestimate the true antimicrobial or antibiofilm potential of these extracts. Extending the incubation time might also increase the inhibitory activity of AFJ and AFBS.

As observed in the present investigation, CHX is capable of suppressing early biofilm formation to a higher extent compared to AFJ or AFBS. In this regard, studies on the suppressive nature of CHX on early biofilms already exist [43,50,60,61]. In our present investigation, susceptibility of all agents declined by increasing biofilm age. This is in line with data published earlier by our group [43].

Anyhow, the introduced model was efficient and can be recommended for further biofilm controlling investigations.

## 5. Conclusions

In the present work, the method of establishing a stable oral ex vivo biofilm from plaque and saliva samples is described. Furthermore, the model was used as an example for observing the efficiency of blackcurrant fruit extracts and CHX in inhibiting ex vivo biofilm growth. It was found that blackcurrant all-fruit juice and CHX have some inhibitory activity, especially on early biofilms. Ex vivo biofilms grown for 24 h were less susceptible.

Following the proposed protocol will ensure the establishment of a polymicrobial biofilm that can be used for further investigations. The model enables stable ex vivo biofilm formation already after 24 to 72 h of cultivation. Therefore, the model can be considered an efficient in vitro tool for studying the efficiency of biofilm-controlling agents.

## Figures and Tables

**Table 1:** Suppression of oral ex vivo biofilm growth in CFU/mL (log_10_). Biofilm age was 1, 4 and 24 h. Biofilms were exposed to AFJ (all-fruit juice), AFBS (alcoholic fraction from berry skins) and CHX (0.2% chlorhexidine, positive control) for 1 and 3 min. Reduction in biofilm growth was compared to the negative control (PBS).

Ex Vivo Biofilm Age	1 h				4 h				24 h			
Exposure Time	1 min		3 min		1 min		3 min		1 min		3 min	
AFJ	−1.84	*p* < 0.001	−1.93	*p* < 0.001	−2.05	*p* < 0.001	−2.21	*p* < 0.001	−0.79	*p* = 0.023	−0.83	*p* = 0.035
AFBS	−0.59	*p* = 0.002	−1.00	*p* = 0.001	−0.80	*p* = 0.019	−0.69	*p* = 0.023	−0.29	*p* = 0.143	−0.26	*p* = 0.147
CHX	−2.55	*p* < 0.001	−3.23	*p* < 0.001	−1.30	*p* = 0.002	−1.42	*p* < 0.001	−0.32	*p* = 0.075	−0.44	*p* = 0.063

## Data Availability

The original contributions presented in this study are included in the article. Further inquiries can be directed to the corresponding author.

## Abbreviations

The following abbreviations are used in this manuscript:

AFJ	All fruit juice
AFBS	Alcoholic fraction berry skins
CFU	Colony forming units
CHX	Chlorhexidine
PBS	Phosphate-buffered saline
